# Modelling within-Host Spatiotemporal Dynamics of Invasive Bacterial Disease

**DOI:** 10.1371/journal.pbio.0060074

**Published:** 2008-04-08

**Authors:** Andrew J Grant, Olivier Restif, Trevelyan J McKinley, Mark Sheppard, Duncan J Maskell, Pietro Mastroeni

**Affiliations:** 1 Department of Veterinary Medicine, University of Cambridge, Cambridge, United Kingdom; 2 Cambridge Infectious Diseases Consortium, University of Cambridge, Cambridge, United Kingdom; Stanford University, United States of America

## Abstract

Mechanistic determinants of bacterial growth, death, and spread within mammalian hosts cannot be fully resolved studying a single bacterial population. They are also currently poorly understood. Here, we report on the application of sophisticated experimental approaches to map spatiotemporal population dynamics of bacteria during an infection. We analyzed heterogeneous traits of simultaneous infections with tagged *Salmonella enterica* populations (wild-type isogenic tagged strains [WITS]) in wild-type and gene-targeted mice. WITS are phenotypically identical but can be distinguished and enumerated by quantitative PCR, making it possible, using probabilistic models, to estimate bacterial death rate based on the disappearance of strains through time. This multidisciplinary approach allowed us to establish the timing, relative occurrence, and immune control of key infection parameters in a true host–pathogen combination. Our analyses support a model in which shortly after infection, concomitant death and rapid bacterial replication lead to the establishment of independent bacterial subpopulations in different organs, a process controlled by host antimicrobial mechanisms. Later, decreased microbial mortality leads to an exponential increase in the number of bacteria that spread locally, with subsequent mixing of bacteria between organs via bacteraemia and further stochastic selection. This approach provides us with an unprecedented outlook on the pathogenesis of S. enterica infections, illustrating the complex spatial and stochastic effects that drive an infectious disease. The application of the novel method that we present in appropriate and diverse host–pathogen combinations, together with modelling of the data that result, will facilitate a comprehensive view of the spatial and stochastic nature of within-host dynamics.

## Author Summary

Global patterns and mechanistic determinants of bacterial spread in mammalian organisms are difficult to obtain through numerical and topographical mapping of a single bacterial population. Appreciation of the true pathogenetic events during infections needs to be based on the understanding of the fine interactions that control the infection dynamics of individual subpopulations in the same host. We have used molecular techniques to tag individually otherwise identical subpopulations of bacteria. We have used these bacteria, called wild-type isogenic tagged strains (WITS), in simultaneous infections in the same animal to gather insights into the patterns of spread of individual subpopulations of bacteria in the tissues and interactions between bacteria and phagocytes. Combining numerical fluctuation in the WITS populations with mathematical modelling and statistical analysis, we have gathered data on the relative occurrence of bacterial growth and death in different phases of the disease process. Our analyses support a model in which shortly after infection, concomitant death and rapid bacterial replication lead to the establishment of independent bacterial subpopulations in different organs. Later, decreased microbial mortality leads to an exponential increase in the number of bacteria that spread locally, with subsequent mixing of bacteria between organs. The work illustrates the importance of unravelling heterogeneous traits of infections to reconstruct and understand the true nature of the global disease process.

## Abstract 3

Genetically identical bacterial strains reveal the population dynamics and interactions of subpopulations of bacteria with the host's immune system in vivo during infection.

## Introduction

Central to a complete understanding of any disease is the ability to integrate information from different scales into a coherent model that fully explains the disease process [1]. One challenge that remains is how to move from our ever more detailed understanding of cellular and molecular microbiology in artificial laboratory systems, towards an explanation of the dynamics of pathogen survival and growth in a whole-animal infection model [2]. A promising approach relies on concepts and mathematical models from population ecology to derive quantitative information on within-host bacterial dynamics from experimental data [3,4]. However, development of an appreciation of within-host dynamics has been hampered by the difficulty of identifying and observing directly, within tissues, the multiple key variables that underlie the infection process. Numerical variations in total microbial load in bacterial infections and the effects of host factors or therapeutic intervention on these variations are usually followed in vivo by counting the number of colony-forming units (CFUs) present in infected organs and plotting this through time. This provides an overarching idea of the global numerical variation in the bacterial population in the organ as a whole, but gives no information about the fine structure of the population in vivo. At one extreme, all bacteria might display identical growth in all infected cells. At the other extreme, only one clone of bacteria might be growing very rapidly, with all others not growing or growing very slowly. Both these scenarios could lead to the same overall growth dynamics at the organ level. However, the biology underlying them would be very different, having major consequences for understanding the most appropriate way to manipulate the immune system and/or antimicrobial drug regimes to combat these infections. Attempts have been made to measure the actual division rate of bacteria in vivo (reviewed by [2]): they typically rely on introducing nonreplicating elements (e.g., phages, plasmids, or temperature-sensitive mutants) into the bacteria. However, their widespread use has been hindered by the sensitivity of foreign elements to experimental conditions. In addition, the extent to which those methods disrupt the phenotype of bacteria and the immune response has not been assessed.

The aim of this study was to obtain a comprehensive picture of in vivo bacterial population dynamics by unravelling the variations in basic demographic processes that drive spatiotemporal events. To this aim, it was necessary to use (1) a host–pathogen system already established as a model for bacterial dynamics, (2) a reliable marker for bacterial division or death that does not modify the infection, and (3) an analytical platform that allowed us to elicit information about unobserved processes from observed data. In this study, we used Salmonella enterica serovar Typhimurium infections in mice, a well-documented, genetically tractable system and a natural host–pathogen interaction with defined infection dynamics amenable to robust statistical analysis. S. enterica infections of mice are a good model for human typhoid fever, for other invasive salmonelloses, and for infections with intracellular pathogens in general.

**Figure 1:**
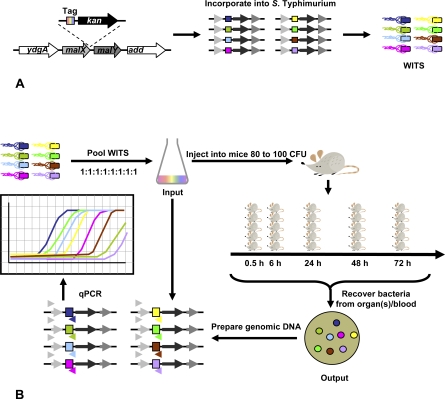
A Cartoon Detailing the Construction of the WITS and the Experimental Design (A) To investigate the in vivo population dynamics of S. enterica during systemic infection, we generated a series of WITS. These are S. enterica strains each carrying a different 40-bp ([NK]_20_ : N = A, C, G, or T; K = G or T) signature tag [29] in precisely the same noncoding region of the chromosome (between two pseudogenes *malX* and *malY*). Each mutant differs from the others only in that it carries a different tag, thus there should be no selective advantage between the different mutants. (B) Mice were inoculated i.v. with approximately 80 to 100 CFU of *S.* Typhimurium, being approximately equal CFU of each of eight WITS. At 0.5, 6, 24, 48, and 72 h p.i., groups of mice were killed, and the livers, spleens, “other organs” (kidneys, heart, lungs, and mesenteric lymph nodes), and blood were cultured for the presence of bacteria. All bacterial colonies were scraped from the agar plates, and chromosomal DNA was purified using standard commercial kits. The S. enterica population structure in individual organs at different times after i.v. inoculation was detected using quantitative PCR. Each biological sample was assessed by qPCR using each of the eight primer pair combinations, the number of copies of each WITS chromosomal DNA per reaction (biological sample) was calculated from standard curves for each of the WITS.

We report an integrated approach to investigate bacterial growth dynamics over the first few days of an acute murine *Salmonella* infection, which relies on tracing tagged subpopulations of otherwise identical bacteria through infected animals and their organs. Previous studies [5–7] have exploited coinfections of single hosts with several strains of a given pathogen as a powerful tool to explore population dynamics during infection; however, such studies are limited by three main factors: the number of strains that can be distinguished inside the host, the limit of detection/discrimination of strains, and the potential phenotypic variations associated with genetic diversity. To overcome these limitations, we generated eight wild-type isogenic tagged strains (WITS); phenotypically identical bacterial strains each carrying a different 40-bp DNA signature tag in the same noncoding region of the chromosome (Figure 1A) with no interstrain difference in in vitro or in vivo growth rates. Using inocula consisting of mixtures of low numbers of each tagged strain, we monitored the dynamics of each subpopulation at different body sites throughout the course of a systemic infection.

We address three specific questions about the dynamics of infection: how does an acute bacterial infection replicate and grow in infected organs? Does the spread of infection within and between organs result from homogeneous bacterial growth, or from expansion of subpopulations? How do components of the immune response affect spatiotemporal bacterial population dynamics? We used depletion of tagged diversity as a proxy for bacterial death, and spatiotemporal heterogeneity as a proxy for bacterial transfer between organs as a function of time postinoculation. This allowed the development of mathematical models to dissect the determinants of net growth at different infection stages, and the mapping of local (intraorgan) versus systemic spread of each bacterial subpopulation. This work illustrates that to understand the basic idiosyncrasies of infection requires a disaggregation of the global disease dynamics and awareness of the separate infection dynamics of subpopulations within the same host. This approach is suitable for many animal infection models; evaluating infection dynamics in these terms will provide a paradigm shift in our understanding of the whole animal infection process.

## Results

### Using WITS to Determine the Within-Host Growth/Death Rate of S. enterica during an Infection

**Figure 2:**
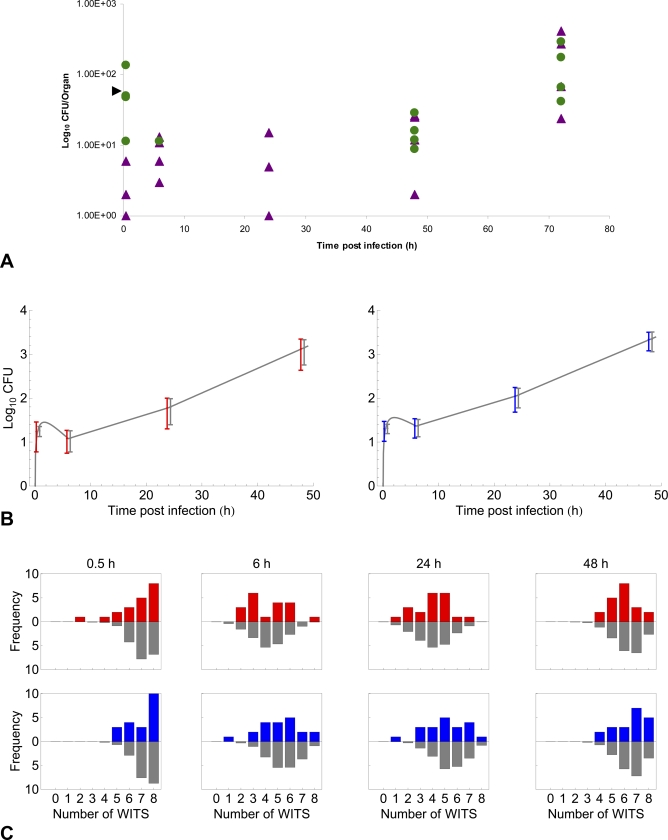
CFU and WITS Frequencies in the Organs of Infected Mice (A) Log_10_ CFU in “other organs” (purple triangle) and blood (green circle) at 0.5, 6, 24, 48 h (*n* = 5), and 72 h p.i. (*n* = 4); the arrow indicates inoculum size. (B) Log_10_ CFU in liver (red) and spleen (blue) (*n* = 20 per time point), results expressed as mean log_10_ viable count ± standard deviation. Lines (grey) indicate deterministic models; (grey) error bars indicate stochastic simulations (described in Protocol S1) expressed as mean ± standard deviation. (C) Frequency of WITS recovered from liver (red) and spleen (blue) compared to distributions of WITS as predicted from 1,000 stochastic simulations (grey).

**Figure 3:**
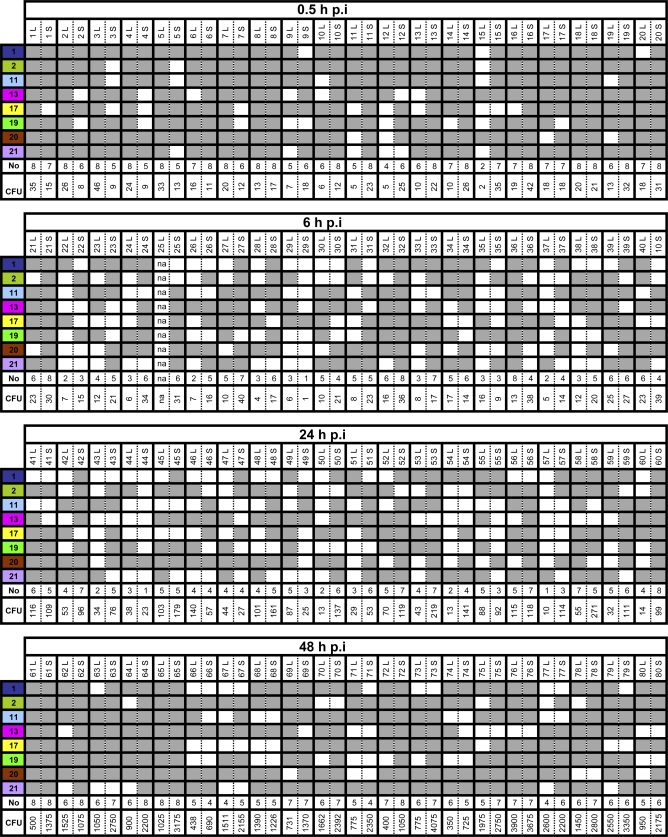
Presence and Absence of WITS in Individual Mice WITS presence (grey box), or absence (white box) in the liver (L) or spleen (S) of individual mice (*n* = 20 per time point) at 0.5, 6, 24, and 48 h p.i. The individual WITS are indicated by coloured boxes (dark blue = WITS 1, olive green = WITS 2, light blue = WITS 11, pink = WITS 13, yellow = WITS 17, bright green = WITS 19, brown = WITS 20, and lavender = WITS 21). In addition, the number of WITS (No) and CFU (CFU) in each organ are indicated.

**Figure 4:**
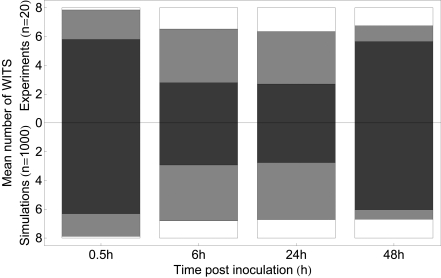
Comparison of the Observed and Predicted (Model) Frequency and Colocalisation of WITS in the Organs Showing the mean number of WITS present in the spleen and liver (dark grey), one organ only (light grey), and neither organ (white). Experimental data are presented above the horizontal time line. The WITS frequencies as predicted by the modelling are presented below the horizontal time line (described in detail in Protocol S1, Models Section 3).

**Figure 5:**
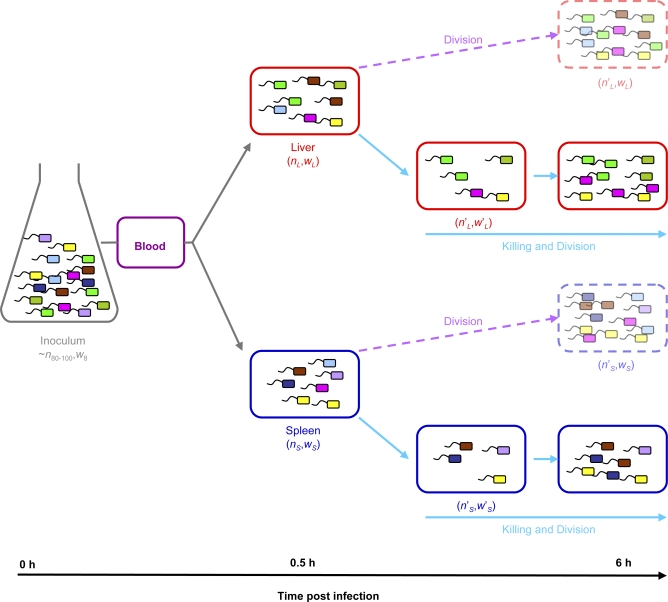
A Schematic Diagram Summarizing the Initial Dynamics of Systemic S. enterica Infections During the early stages of infection, bacteria move from the blood into the spleen and the liver (CFU in each organ denoted by *n_S_* and *n_L_*, respectively), representing a frequency of WITS in the spleen (*w_S_*) and liver (*w_L_*). WITS frequency at 0.5 h p.i. is consistent with stochastic removal of bacteria from the blood by the liver and spleen. Between 0.5 and 6 h p.i., bacterial growth and death occur in parallel, leading to the disappearance of individual subpopulations and the expansion of others. If bacterial death did not occur, the frequency of WITS per organ at 0.5 and 6 h would be comparable (dashed line and faded shading).

As a first step in our study, we monitored the growth kinetics of the WITS in the systemic compartment of mice after intravenous (i.v.) injection (Figure 1B). Total bacterial loads in the different organs are shown in Figure 2A and 2B. Between 0.5 and 6 h postinfection (p.i.). we observed blood clearance, a slight decrease in bacterial numbers in the liver, and a slight increase in bacterial numbers in the spleen. This was followed by exponential bacterial growth at the expected rate of approximately 10-fold increase per day. Low bacterial numbers persisted in the “other organs” throughout, and a resurgence of bacteraemia occurred from 48 h p.i. onwards. To determine the true variation in net bacterial growth rates in the tissues, we monitored bacterial division and death rates in infected tissues to assess whether bacteria started dividing in the spleen and liver within the first 6 h p.i. Using quantitative PCR (qPCR), which in this application provides a discrimination range of at least 10^4^-fold, we analysed the S. enterica WITS population structure in individual organs (Figure 2C). Shortly after inoculation (0.5 h), the qualitative population structure of the WITS between spleen and liver in each animal was not homogeneous (Figures 2C and 3), reflecting low bacterial numbers present in each organ. At 6 h p.i., the WITS population structure remained heterogeneous between the two organs (Figures 2C and 3); however, the frequencies of individual WITS recoverable from each organ were lower than those observed at the earlier time point of 0.5 h p.i. (Figures 3 and 4). Observed WITS distributions enabled us to reject the hypothesis that bacteria have not started dividing by 6 h p.i.; the combinations of bacterial counts and WITS frequencies in the spleens and livers of individual mice at 6 h p.i. were incompatible with random sampling from the initial inoculum (Figures S1 and S2; Protocol S1, Section 1). This indicates that in the first 6 h p.i., heterogeneous bacterial division and death occur in parallel, leading to the disappearance of some bacterial subpopulations and the expansion of others (Figure 5). Thus, the prevalence of individual bacterial subpopulations in an animal originally infected with genetically identical bacteria starts from the very early stages of the infection process.

### The WITS Indicate Bacterial Growth and Death in Early Systemic Infection

We fitted a first mathematical model to the observed number of CFUs in the organs, to estimate (1) initial rates of transfer from the blood into the organs and (2) net growth rates in the first 6 h of infection (Equation 1 in Materials and Methods; Figure S3; Protocol S1, Section 2a). The model suggests that the average 93 bacteria of the inoculum lodge in the spleen and liver within approximately 4 h, and that densities subsequently decrease at rates corresponding to a 50% drop every 5 h in the spleen and every 3 h in the liver. To distinguish between bacterial divisions and death within the first 6 h p.i., we generated another model, which uses a branching process to track the number of copies of an individual WITS population in a given organ (Equation 2; Protocol S1, Section 3). To summarize, at 6 h p.i., we matched the probability of a WITS being absent from an organ in the model with the proportion of WITS found missing across all mice. It is worth pointing out here that the observed decrease in WITS diversity between 0.5 and 6 h p.i. could in theory be due to either bacterial death or migration to other organs or tissues. Actually, we were able to dismiss the latter hypothesis by comparing the WITS found in the liver and spleen within each animal; the high heterogeneity between the two organs at 6 and 24 h p.i. (Figure 4) could not be maintained if there was any bacterial migration between them during that time (Protocol S1, Section 3). We discovered that the slow decline in bacterial numbers during the first 6 h p.i., is caused by the combined occurrence of high division rates (doubling times around 1.7 h in both organs) and higher death rates (half-lives around 1.1 h). The model also allows us to determine the likely maximum duration of this process and predicts that it must cease within 7 h p.i. In fact, if both high division and death rates extended beyond 7 h, the numbers of WITS in the organs would keep decreasing, at odds with the steady WITS distributions observed experimentally at 24 h. Therefore, it is possible to predict that a “switch” from the early phase of active bacterial killing to a phase of low bacterial killing and exponential growth must occur in the spleen and liver within 7 h p.i. (Figures S4–S6, Table S4; Protocol S1, Models 2b). The bacterial dynamics arising from this combination of migration from the blood to the organs, rapid divisions and killing, can be simulated using our mathematical model (grey curve on Figure 2B). The peak of bacterial density in the organs around 2 h p.i. occurs when killing balances immigration and division.

### The WITS Indicate Independent Infections in Systemic Organs during the First 24 h Postinfection

During this second phase of the infection (6 to 24 h p.i.), bacterial numbers increased exponentially; we have previously shown that this is paralleled by a similar increase in the number of infected host cells [4,5]. The frequency of individual WITS in the spleen and liver of each animal remained almost constant (Figures 2C and 3). Our model shows that bacterial mortality in the organs must be very low during this period, so that observed division rates (doubling times around 8 h in both organs) must decrease to reduce the overall observed bacterial growth (3- to 4-fold decrease compared to phase one, 0.5 to 6 h p.i.) (Protocol S1, Section 3). The analysis of WITS frequency between organs (Figure 3) reveals that the observed differences between the liver and the spleen remain different between 6 and 24 h p.i. (Figures 2C and 4). Consequently, the hepatic and splenic subpopulations must grow independently without mixing for at least 24 h, with negligible mortality after 6 h. In summary, the models capture the observed data and predict an initial phase that lasts for at most 6–7 h and is characterised by high division rates and even higher death rates of bacteria in both the spleen and liver, and a second phase that is characterised by very low bacterial death rate, (possibly zero), and moderate division rates in both organs. Thus, we can conclude that net growth rate in the tissues is governed by the bacteria growing at a controlled rate as the host restrains bacterial division, and not by killing of the bacteria. The data also indicate that during the early stages of the exponential growth phase, bacterial subpopulations remain spatially separated, growing and spreading from cell to cell locally [4,5]; they do not escape to other organs.

### The WITS Indicate Local and Then Systemic Haematogenous Spread during Infection

At 48 h p.i., the frequency of individual WITS recovered from the spleen and liver increased and became more homogeneous between the two organs within each mouse (Figures 2C and 3), indicating that the infection had entered a third phase, characterised by systemic bacterial spread and mixing of WITS populations between distant body sites. The high frequency of WITS recovered at 48 h p.i. suggests that bacterial mortality had not increased from 24 h p.i. Systemic dissemination of WITS correlated with the appearance of WITS in the blood. A simple model for global bacterial dynamics (ignoring the WITS structure) allowed us to estimate the relative rate of transfer of bacteria from the organs to the blood, around 1% of the bacterial transfer rate from the blood to the organs (Protocol S1, Section 4a). Collectively, the data indicate local spread within each organ early in infection, followed by later systemic haematogenous spread. The systemic spread can be reasonably explained in the light of bacteraemia; however, the exclusive within-organ spread early in infection is intriguing, especially when considering that S. enterica grows and spreads from cell to cell via the extracellular compartment at a constant rate throughout infection [4].

### Stochastic Model of Systemic S. enterica Infection

**Figure 6:**
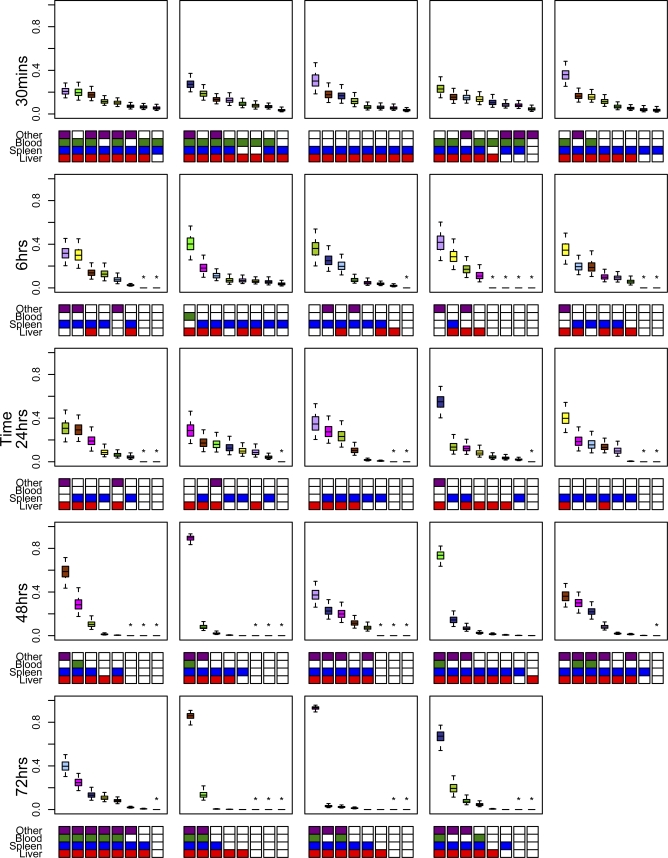
Proportions of WITS Distributions in Mice Results for the predicted proportions of each WITS within individual mice from one experiment, at 0.5, 6, 24, and 48 h p.i. (*n* = 5) and 72 h p.i. (*n* = 4). These were generated from an initial Bayesian linear regression model fitted to log_10_(conc.) against CT value (the point at which the fluorescence crosses the threshold) from the qPCR analysis. Each panel represents a different mouse, with boxes giving the median and quartiles, and the whiskers representing 95% credible intervals centred around the median. Within any panel, the left-hand box plot represents the most frequent WITS, and right-hand box plot the least frequent WITS. Below each panel, the four-line grid indicates the presence (shaded) or absence (white) of each individual WITS (as ordered in the panel directly above) in the different organs: “other organs” (purple), blood (green), spleen (blue), and liver (red). The individual WITS are indicated by coloured boxes (see Figure 3 for a detailed description). Asterisks indicate absence of WITS.

Analysis of the global, proportional population structure of WITS within individual animals demonstrates a skewed subpopulation structure, with increased predominance of typically a couple of WITS within each animal at 48 and 72 h p.i. (Figure 6). The data therefore indicate preferential amplification of subpopulations during the infection process despite the fact that the WITS exhibit no systematic bias in terms of relative “fitness” or ability to colonise the host. The lack of bias is an assumption built into the models and is based on experimental data showing qualitatively, (Figure 6) and quantitatively (*p*-value > 0.97) (Table S5), no evidence of colonisation bias in any of the individual WITS across mice. We now complete our analysis with a stochastic model that encompasses all the processes previously analysed over the first 48 h p.i. and that allows for random variations in the initial conditions (Protocol S1, Section 4.b; Table S6). More precisely, we assumed that the number of copies of each WITS in the inoculum followed a Poisson distribution with mean 11.65, in line with the experimental protocol in which an even mix of the eight WITS was diluted down to an estimated density of 93.22 bacteria per inoculum (mean value from nine independent samples that was determined by plating aliquots of the input inocula onto LB agar plates). We tracked the numbers of WITS in the “virtual” blood, liver, and spleen in a series of 1,000 simulations, and compared the output to experimental observations (Figures 2B, 2C, 4, and S7). The simulations, which rely on demographic stochasticity, reproduced levels of variability compatible with those observed experimentally, except at the earliest time point (0.5 h p.i.), when empirical data were slightly more variable. This could be due to heterogeneities among mice or among experiments or, possibly, to mechanistic detail not included in the model. Although most of our analyses have focused on patterns of presence/absence of WITS, our stochastic model actually keeps track of the number of copies of each WITS in the blood, liver, and spleen. This enabled us to compare, at least qualitatively, the patterns of WITS proportions in the simulations and in the experiments (Figure S7), providing further support for the validity of our approach. The only notable discrepancy related to distributions in the blood at 0.5 and 48 h p.i. where simulations appear to underestimate the level of heterogeneity. This is likely to be due in large part to the small blood sample sizes in the experiments, which may have resulted in the less frequent WITS being systematically underreported.

### Using WITS to Investigate How Immunological Bottlenecks Affect the Distribution of S. enterica during an Infection

**Figure 7:**
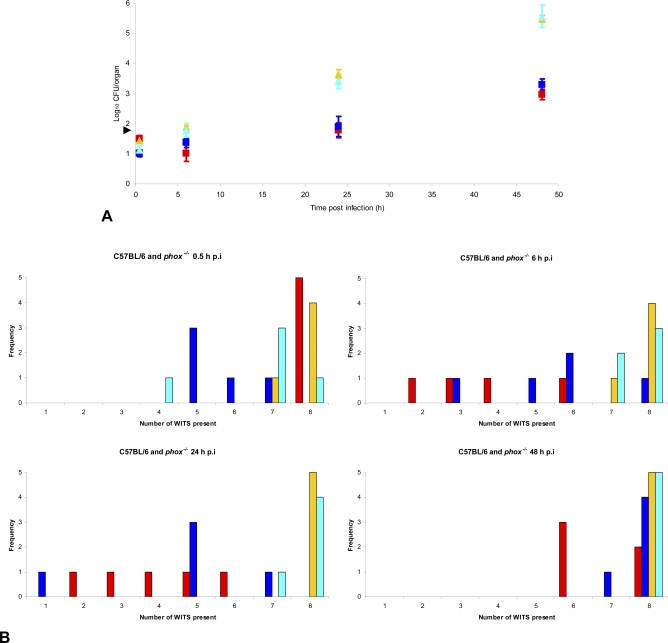
CFU and WITS Frequency in Infected C57BL/6 and *phox^−/−^* Mice *phox^−/−^* mice and congenic wild-type control mice on a C57BL/6 background were infected i.v. with approximately 80 to 100 CFU of *S*. Typhimurium WITS. (A) shows bacterial numbers and (B) the frequency of WITS recovered, from the C57BL/6 liver (red square), C57BL/6 spleen (blue square), *phox^−/−^* liver (golden triangle), and *phox^−/−^* spleen (cyan triangle) at 0.5, 6, 24, and 48 h p.i. Results are expressed as log_10_ viable count ± standard deviation; arrow indicates inoculum.

The approach combining the use of WITS and mathematical analysis of fluctuations in individually tagged bacterial populations within an animal can also be used as a powerful tool to analyse the effects of immunological mechanisms on the population dynamics of infection. As an example, we used WITS infections in *phox^−/−^* mice to investigate the role of NADPH oxidase in controlling S. enterica during systemic infection. Reactive oxygen intermediates (generated via the phagocyte NADPH oxidase, encoded by *phox*) are produced by phagocytes at infection foci to control the growth of intracellular bacteria [8,9]. We analysed fluctuations in total bacterial numbers and WITS population structure throughout the infection. CFU and frequency of WITS in the organs of *phox^−/−^* mice at 0.5 h were comparable to wild-type C57BL/6 mice (Figure 7A and 7B). However, by 6 h, when bacterial load in spleens and livers of wild-type mice had decreased by roughly one quarter, bacterial load in *phox^−/−^* mice had increased over 12-fold. This observation combined with the fact that at 6 h, no WITS were lost in the majority of the *phox^−/^* ^−^ mice, indicates little or no killing in the absence of a functional NADPH oxidase and demonstrates the bactericidal action of NADPH oxidase in these early phases of the disease process. The lack of disappearance of WITS in the *phox^−/−^* mice also indicates that the early in vivo selection of bacterial subpopulations observed here in S. enterica infections is actively mediated by innate immunity mechanisms. The calculated combined mean generation time (spleen and liver) between 6–48 h gives 7.46 h and 3.38 h for wild-type and *phox^−/−^* mice, respectively. The difference in generation time, which occurs in a phase of the infection when bacterial killing is negligible, indicates a bacteriostatic role for NADPH oxidase from 6 h p.i. The data provide an increasing level of resolution in understanding the dynamics of antibacterial functions of phagocytes. In fact, contrary to what was previously postulated [8,9], the reactive molecules generated by NADPH oxidase are highly bactericidal only in the very early stages of infection, becoming bacteriostatic as infection progresses. This analysis illustrates, therefore, that an individual immunological mechanism can have different sequential effects on bacterial population dynamics. This may reflect transmission from resident macrophages to polymorphonuclear neutrophils (PMNs) [10], and consequent differences in intracellular control mechanisms.

### Using Model Frameworks to Make Predictions about the Infection Process

**Figure 8:**
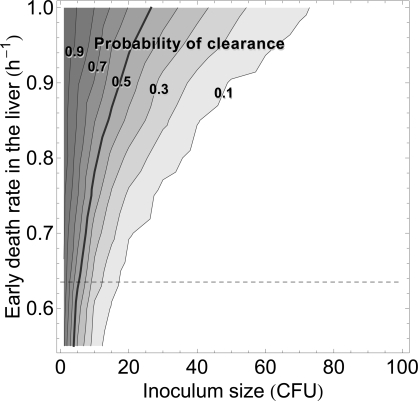
Estimated Probability of Clearance of Infection for Various Inoculum Sizes and Initial Death Rates Probability values shown along the isoclines were estimated as the proportion of stochastic simulations resulting in bacterial clearance. For each combination of inoculum size (horizontal axis) and death rate (vertical axis), we ran 1,000 simulations. Early death rate in the spleen was varied in line with that in the liver (i.e., the difference between the two parameters remained constant). The horizontal dashed line represents the baseline death rates as estimated from the experiments. The thick isocline represents the predicted ID_50_ (i.e., intravenous inoculum size giving a 50% risk of successful infection) read on the horizontal axis as a function of death rate read on the vertical axis. Variation in death rates might be achieved with different mouse strains or following treatment or immunisation. Other parameter values were as estimated from the experimental values.

We can use the model frameworks that we have generated, not only as a resolving tool to increase the power of the biological data, but also to make additional predictions about the infection process. For example, we can consider the probability of clearance of the infection for various inoculum sizes. Our analysis predicts that the 50% infectious dose (ID_50_) for the *S.* Typhimurium WITS is approximately 5 CFU (Figure 8), in line with the 50% lethal dose (LD_50_) of the parent strain, SL1344 for BALB/c mice, fewer than 20 CFU [11]. Figure 8 also suggests that if the initial bacterial death rate in the early phase of infection is increased from 0.64 to 1.0 h^−1^ in the liver and from 0.59 to 0.95 h^−1^ in the spleen, the ID_50_ increases to approximately 25 CFU, thus demonstrating the ability of the models to shed light on the within-host dynamics of bacterial control.

## Discussion

The course and outcome of a S. enterica infection depends upon many host and bacterial factors, and a number of models have been proposed to describe the infection process: the hypothesis of independent action of pathogens [12], the birth–death model [13], and the two-stage model of microbial infection [14]. Results from numerous studies over the last 50 y indicate that the progression of murine salmonellosis can be divided into two different phases, an early phase of exponential *Salmonella* growth in the spleen and liver, and a later septic phase that precedes death [15,16]. The onset of these phases differs according to variations in experimental design and differences in bacterial and host strain.

Bacterial growth, death, and spread in the body are still undetermined aspects of the infection process despite their exquisite relevance to vaccination and treatment. Studies often focus on observation of the disease course at the whole-animal level or in whole organs, and interpret the gross, cumulative effects of many individual interactions and use the mean to represent what is a complex phenomenon. However, an appreciation of population dynamics at the gross level provides almost no information on where individual bacteria locate and how they spread and interact during infection. Simply assessing bacterial load in each animal by plate count is not sufficient or sophisticated enough to allow us to fully understand the infection process. Spatial and functional independence between individual bacterial populations in the same host has been conclusively demonstrated by several studies from our group. Previously, we have shown that during a systemic S. enterica infection, each infectious focus is a separate unit resulting from clonal growth of an individual bacterium, with spatial segregation of bacterial populations and continuous distribution to new phagocytes throughout the infection process [4,5,17]. Thus, understanding the overall dynamics of an infectious disease hinges on the elucidation of the complexity and dimensionality of functional independence and heterogeneity at the subpopulation level in the animal.

In this study, we have used an infection model based on simultaneous administration of tagged subpopulations of otherwise identical bacteria (WITS) in the same animal, to resolve the difficult question of how components of the host immune responses counteract the net growth of the bacterial load in the tissues and to determine the relevance of bactericidal and bacteriostatic mechanisms in these processes. We have shown that shortly after infection, concomitant NADPH oxidase-dependent death and rapid bacterial replication lead to the establishment of independent bacterial subpopulations in different organs. Subsequently, a reduction in bacterial mortality leads to an exponential increase in the number of bacteria that spread locally and independently within each organ (e.g., spleen and liver). During the later stages of infection, different bacterial populations mix between organs via the blood with a further stochastic selection of bacterial subpopulations.

Populations of genetically identical bacterial strains are a very powerful tool to address novel questions on the nature of individual events that determine the global profile of population dynamics during infection. Having developed and validated the use of WITS in the study of the in vivo population biology of *S. enterica,* we are now in the position to exploit these systems to great effect to understand the impact of key host and bacterial factors on the pathogenesis of S. enterica at the level of individual bacterial subpopulations. This also allows us to address the extent to which we can rely on data generated in in vitro cell biology systems to describe what is actually happening in vivo. Understanding the effect of anatomical bottlenecks and individual components of the immune system on local and bacteraemic spread is fundamental in order to tailor appropriate prevention and treatment strategies to different stages and forms of S. enterica diseases in animals and man.

The specific model we have chosen has focused on S. enterica, but it is an approach that can be applied to the study of the population dynamics of many infectious animal diseases. The use of tagged wild-type strains has the potential to transform our understanding of within-host dynamics of pathogen interactions with host cells, to provide the information needed to build mechanistic mathematical models, generate new research hypotheses, and make quantitative predictions. For example, by using combinations of WITS in animals from different genetic backgrounds, in gene-targeted immunodeficient animals, or in previously vaccinated animals, it will be possible to investigate the way in which different components of the innate and acquired immune system control the expansion (net growth rate) or determine the contraction (clearance) of the bacterial load in the tissues. Using combinations of defined bacterial mutants and gene-targeted mice, it will be possible to determine precisely how individual bacterial virulence genes counteract the antimicrobial functions of the host. Moreover, understanding the impact of the immune response and different classes of vaccines on the distribution and population dynamics of pathogens will be instrumental in furthering the development of preventive measures and drug therapies.

## Materials and Methods

### Bacterial strains, media, and growth conditions.

All bacterial strains used in this study are listed in Table S1. The Escherichia coli strain DH5α was used for gene cloning, unless otherwise indicated. S. enterica serovar Typhimurium strain JH3010 [18] is a virulent wild-type strain, derived from SL1344, which has an LD_50_ of fewer than 20 CFU for BALB/c mice [11]. E. coli strains were grown for 16 h in Luria-Bertani (LB) broth at 37 °C; cultures were shaken at 220 rpm. S. enterica strains were grown for 16 h as a stationary culture at 37 °C in LB broth. Where necessary, media were supplemented with the appropriate antibiotic for selection (ampicillin, 100 μg/ml, kanamycin 50 μg/ml, and chloramphenicol 10 μg/ml). S. enterica were diluted in phosphate-buffered saline (PBS) prior to i.v. inoculation. Long-term storage of bacteria was at −80 °C in Microbank vials (Prolab Diagnostics). Preparation of electrocompetent E. coli and S. enterica cells and transformations were performed as previously described [19]. In vitro growth rates of WITS in LB broth were determined by optical density reading of LB broth cultures, serially diluted at each time point, and also plated to obtain the number of CFU per millilitre.

### Animals.

All aspects of animal procedures were approved by the local ethical committee and performed according to UK law. *phox^−/−^* mice were bred in the Cambridge animal unit from breeding pairs generously provided by Prof. Jennie Blackwell [20]. Female C57BL/6 mice were purchased from Harlan Olac Ltd., Blackthorn, Bicester, UK, and used when over 8 wk of age.

### Enumeration of viable *Salmonella* in tissues and blood.

Approximately 0.4 ml of blood was obtained from the tail vein. Mice were killed by cervical dislocation; the liver and spleen were removed and individually homogenised in a Seward Stomacher 80 Biomaster (Seward) in 5 ml of distilled water. “Other organs” (lungs, heart, kidneys, and mesenteric lymph nodes) were removed and homogenized together, using an Ultra-Turrax T25 blender in 5 ml of distilled water. Viable bacterial counts from the whole organ(s) or blood were assayed on plates of LB agar supplemented with antibiotics where necessary for selection. To obtain the estimated total number of CFU in the blood of an animal, the number of CFU per volume in the sample obtained was corrected by assuming a total circulating blood volume of 2 ml.

### Recombinant DNA techniques.

Standard methods were used for molecular cloning [21]. Chromosomal and plasmid DNA purifications were performed using commercial kits following the manufacturers' instructions (QIAGEN). Routine DNA modifications, including restriction endonuclease digestion of DNA, modifications of DNA, and ligations, were carried out as per manufacturers' instructions (Promega, Invitrogen, Roche, and New England Bioloabs). DNA concentration and purity were measured using a Nanodrop ND-1000 spectrophotometer.

### Oligonucleotides and PCR.

The sequences of primers used in this study are listed in Table S2 and were purchased from Sigma (Sigma-Genosys). Q-PCR primers were designed using Primer3, freely available at http://frodo.wi.mit.edu/, employing, where possible, the parameters of Inglis and Kalischuk [22] (product of 105–125 bases, length of primer 18–25 bases, melting temp 58–60 °C, GC% 50–60, no 3′ T, no more than two G or C residues in the last five bases at the 3′ end). PCRs were carried out as described previously [16]. All PCRs were performed in 25-μl reaction volumes in 0.2-ml Eppendorf tubes in a PerkinElmer Gene Amp 2400 thermal cycler. Reactions contained 200 μM dNTPs, 2 mM Mg^2+^, 0.01 volumes of Proof Start DNA polymerase (QIAGEN; 2.5 U μl^−1^), 0.1 volumes polymerase buffer (10×), 1 μM forward and reverse primers, and template DNA (∼50-ng plasmid DNA or ∼100-ng chromosomal DNA). Typical thermal cycler conditions were 94 °C for 5 min, 30 cycles of 94 °C for 1 min, 55 °C for 1 min, and 72 °C for 1 min, followed by a final extension of 72 °C for 10 min.

### Quantitative PCR.

All qPCRs were performed in 25-μl reaction volumes in 0.1-ml tubes in a Corbett Research RG3000. Reactions contained 12.5 μl of QuantiTect SYBR Green PCR Kit reagent (QIAGEN), 7 μl of RNase-free water, 25 μM forward and reverse primers, and 5 μl of template DNA (∼1–10 ng per reaction = ∼10^5–^10^6^ copies of DNA/reaction). Reaction conditions were 95 °C for 15 min, 40 cycles of 94 °C for 15 sec, 61 °C for 30 sec, and 72 °C for 20 sec. It was not possible to perform a full standard curve for each primer pair on every rotor; however, individual standards were included on each rotor run to ensure that the values obtained were in the range expected. (For detailed description of qPCR method, see Protocol S1, Materials and Methods section). For scoring WITS as present/absent, a threshold value was determined with reference to the background values obtained with the standards for a given batch of QuantiTect SYBR Green PCR Kit reagent. Typically, WITS were scored as present if the value was above 10^2^ copies of DNA/reaction. However, for one experiment when the typical amount of DNA used in each reaction was greater than 10^7^ copies, the cutoff value was taken as 10^3^ copies of DNA/reaction due to the increased background.

### Plasmids.

All of the plasmids used in this study are listed in Table S3. pBADλred [23] was induced with 0.2% l-arabinose. pAJG300 was generated by PCR amplification of part of *ydgA* and *malX* with primers ajg350 and ajg351 using SL1344 chromosomal DNA as template. The product was cloned into the EcoRI and BamHI sites of pUC19 as an EcoRI-BamHI fragment. The *rpsM* promoter was cloned into the BamHI and XbaI sites of pAJG300 as a BamHI-XbaI fragment generated by PCR amplification from SL1344 chromosomal DNA using the primers ajg352 and ajg353 (BamHI and XbaI sites incorporated into primers) to generate pAJG301. The *gfp* ^+^ gene was cloned into the NheI and PstI sites of pAJG301 as an NheI-PstI fragment generated by PCR amplification from pWH1012gfp^+^ [24] using the primers ajg354 and ajg355 (NheI and PstI sites incorporated into primers) to generate pAJG303. PCR amplification of part of *malY* and part of *add* was performed with primers ajg356 and ajg357 using SL1344 chromosomal DNA as template, and cloned into the PstI and SphI sites of pAJG303 as a PstI-SphI fragment, generating pAJG309. After XbaI digestion of pAJG309, an approximately 2.9-kbp fragment, containing *ydgA,malX,rpsM,gfp^+^,malY,add*, was cloned into XbaI-digested pDS132 [25] to generate pAJG315. The tags were generated by PCR amplification from DNA generously donated by Prof. David Holden [26]. DNA sequencing (conducted by GeneService, Cambridge, UK) was used to confirm that the plasmids had individually identifiable (NK)_20_ tag sequences. Individually tagged constructs were constructed as follows: a kanamycin resistance cassette was PCR amplified from pACYC184 using primers ajg400–420 (each forward primer contained a unique 40-bp tag) and ajg425. The resulting products were digested with BglII and SpeI, and cloned into BglII-SpeI–digested pAJG315, generating the individually tagged constructs.

### Chromosomal integration of tags in *Salmonella* strains.

Individually tagged kanamycin constructs were integrated onto the chromosome of *S.* Typhimurium strain JH3016 [18] using a modification of the ET cloning procedure [27,28] as previously described [23]. A fragment containing an individual 40-bp tag and the kanamycin resistance cassette was amplified from the individually tagged constructs, using the primers ajg474, 475, 481, 482, 485, 487, 488, 489, and ajg465. Approx 1 μg of each linear PCR product was used for integration onto the chromosome using a modification of the Lambda Red method [29], as previously detailed [23]. Transformants were verified by plating onto selective media. Loss of the pBADλred helper plasmid was essentially as previously described [18], using MAST ID Intralactam circles (MAST Diagnostics) to screen for the absence of beta-lactamase in bacterial colonies. Loss of the helper plasmid was also confirmed by a negative PCR result using primers to the *bla* gene (ajg516 and ajg517). The resultant mutants, WITS with a chromosomally located individually tagged kanamycin cassette were confirmed by PCR using a primer designed to the respective unique tag and primer ajg469 designed to *ydgA*, away from the integration site (unpublished data). Additionally, candidates were verified by Southern hybridization (unpublished data).

### Mathematical models for bacterial dynamics.

We designed two sets of mathematical models in order to describe the dynamics of the system and estimate six key parameters: bacterial division rates in the liver and the spleen, bacterial death rates in the liver and the spleen, and transfer rates from the blood to the liver and the spleen. In all the analyses, unless specified otherwise, we assume that the inoculum follows a Poisson distribution with mean 93.22 bacteria, and that the number of CFU of each WITS follows a Poisson distribution with mean 11.65 (i.e., 93.22/8). This was guided by the experimental protocol, in which an initial mix with (approximately) equal CFU of each WITS was diluted to obtain an expected number of 80 to 100 bacteria. The actual bacterial numbers inoculated into each mouse could not be measured before the infection, but in nine independent aliquots from the input inocula, we obtained a mean of 93.22 CFU and a standard deviation of 11.31 CFU. We ignored bacteria demography in the blood, and as explained in the Results section, we dismissed transfer from the organs back into the blood until after 24 h p.i. after analysing experimental evidence. Based on the observation that most bacteria can be found at any time point in either blood, the spleen, or the liver, we only included these three compartments in our models. Because the data pertaining to the “other organs” were scarce and composed of very low numbers, their inclusion in our models would have added unnecessary complexity and would have almost certainly led to very similar results. Bacterial dynamics were described using simple differential equations classically used for population dynamics. Importantly, we assumed that the parameters of the model could vary during the course of infection (leading us to identify three main phases, see Results and Protocol S1), but at any given time, they did not differ among bacteria within each organ.

Here, we describe overall bacterial dynamics (regardless of WITS) as three interconnected populations: *n_B_*, *n_L_*, and *n_S_* represent the expected bacterial numbers of bacteria in the blood, liver, and spleen, respectively. This leads to the following set of equations:

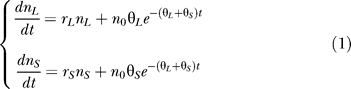
where *r_L_* and *r_S_* are the net growth rates of bacteria in the liver and the spleen (we cannot distinguish divisions from deaths until we take the WITS into account), *θ_L_* and *θ_S_* are the transfer rates from the blood to the liver and the spleen, and *n*_0_ is the inoculum size. So, in the equations above, 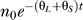 represents the expected number of bacteria left in the blood at time *t*. As explained in Protocol S1, Section 2, we then fitted this model to the data to estimate the four parameters over different time periods.

In order to estimate the underlying division and death rates, we used a second model that describes the probability distributions of the numbers of copies of a single WITS in the liver, spleen, and blood. Indeed, since the eight WITS are identical and independent, there is no need to track them all simultaneously. We denoted by *q^L^_m,n_*(*t*) the probability that *m* and *n* copies of any given WITS are present in the blood and in the liver, respectively, at time *t*, and by *q^S^_m,n_*(*t*) the probability that *m* and *n* copies are present in the blood and in the spleen, respectively, at time *t*. The division rates are named *λ_L_* and *λ_S_*, and the death rates *μ_L_* and *μ_S_* (in the liver and the spleen, respectively). The dynamics for the liver in the early phase of infection are governed by the following equations:

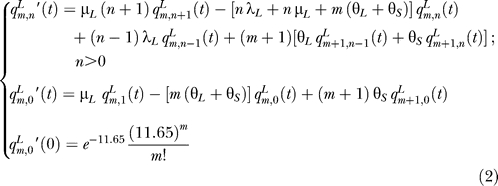



The last equation represents the initial condition, assuming that the inoculum size follows a Poisson distribution of mean 11.65 for each WITS. The equations for the spleen can be obtained by swapping *L* and *S*. Because we had previously estimated the transfer rates and the net growth rates *r_L_* = λ*_L_* − μ*_L_* and *r_S_* = λ*_S_* − μ*_S_*, we estimated the death rates in each organ by fitting this second model to the WITS data. In brief, we used the model to assess the probability of extinction of a single WITS at 6 and 24 h p.i., from which we derived the probability distribution of the number of WITS present in each organ at those two time points. We then fitted the expected mean to the observed mean number of WITS averaged across all mice. (See Protocol S1 for more details).

## Supporting Information

Figure S1 Schematic to Illustrate the Sampling Model (A) Two random samples (spleen and liver) are taken from the initial inoculum: given the sample sizes *n_L_* and *n_S_*, the model returns the frequency of WITS *w_L_* and *w_S_*.
(B) If divisions occur in the organs before the observation is made, then the number of bacteria increases from *n* to *n*′, while the number of WITS *w* remains constant; in other words, the observed number of WITS should be lower than expected under the null hypothesis given the observed sample size *n*.
(1.2 MB TIF)

Figure S2 Model to Test for Bacterial Growth and Death during the Early Stages of Infection The figure indicates the probability of obtaining, at most, the observed frequency of WITS *w_L_* and *w_S_* given the bacterial counts *n_L_* and *n_S_* under the null hypothesis (no bacterial divisions) in individual mice, at 0.5 and 6 h p.i. A single asterisk (*) denotes significance at *p* ≤ 0.05.
(2.7 MB TIF)

Figure S3 Bacterial Growth in the Liver and Spleen—Settling in the Organs Numerical estimates of transfer rates from the blood (*θ_L_* [red dashed line] into the liver and *θ_S_* [blue dashed line] into the spleen) and initial net growth rates *r_L_*_1_ (red line) and *r_S_*_1_ (blue line) based on average bacterial counts at 0.5 and 6 h p.i., plotted against the (unknown) inoculum size *n*_0_. The horizontal black dashed line shows the expected average inoculum size in the experiments (93.22 CFU)
(1.0 MB TIF)

Figure S4 Bacterial Growth in the Liver and Spleen Numerical estimates of parameters (A) *r_L_*_1_, (liver, red) and *r_S_*_1_ (spleen, blue), and (B) *θ_L_* (red) and *θ_S_* (blue) plotted against time of switch *χ* and inoculum size *n*_0_.
(16.6 MB TIF)

Figure S5 Bacterial Growth in the Liver and Spleen Numerical estimates of the growth rates in the second phase of infection (6–24 h p.i.) of infection, *r_L2_* (liver, red) and (spleen, blue) *r_S2_*, plotted against time of switch *χ* and inoculum size *n*_0_.
(10.7 MB TIF)

Figure S6 Probability of Absence of a Single WITS, as Estimated from the Model up to 7 h p.i. Probability is shown (A) in the liver and (B) in the spleen. The arrows indicate the 95% confidence intervals on the observed proportions of missing WITS in each organ at (a) 0.5, (b) 6, and (c) 24 h p.i. If the early dynamics (with high division and death rates) extend for more than 7 h, the model predicts that the proportion of WITS lost will exceed that observed at 24 h p.i (arrow c).
(2.4 MB TIF)

Figure S7 Comparison of WITS Distributions, between Experimental and Stochastic Simulation Data Distributions of WITS proportions per compartment (columns) and time point (rows).
(A) From one experiment (Figure 6), the mean proportions of the eight WITS in each organ were ranked in decreasing order in each of the five mice available at each time point. Box plots represent the distributions of these frequencies across five mice. Within any panel, the left-hand box plot represents the most frequent WITS and right-hand box plot the least frequent WITS.
(B) We performed a similar analysis with the results of 1,000 stochastic simulations. WITS were ranked in each compartment in each simulation according to their proportions.
(3.2 MB TIF)

Protocol S1 Details of Model Constructions and Statistical Analyses (105 KB DOC)

Table S1 Bacterial Strains Used in the Study ^a^ S. enterica strain SL1344 was derived from strain 4/74, which was isolated from a calf bowel.
^b^The *gfp* gene fusion was inserted at the *putPA* locus at positions 1,210,040 to 1,211,657 in the LT2 genome. Φ indicates a transcriptional gene fusion.
^c^The individual *tag::kan* cassette was inserted at the *malXY* locus at base 1,678,843 in the SL1344 genome (http://www.sanger.ac.uk/Projects/Salmonella/).
(15 KB XLS)

Table S2 Oligonucleotides Used in the Study (18 KB XLS)

Table S3 Plasmids Used in the Study (16 KB XLS)

Table S4 Parameters Used in the Models Numerical values (expressed in h^−1^) estimated from the experimental data, assuming transitions at 6, 24, and 36 h p.i. The values that change at each transition are indicated by an up or down arrow.
(15 KB XLS)

Table S5 There Is No Apparent Difference in Relative Fitness between the WITS In order to detect any systematic bias in terms of relative fitness of the WITS, two formal tests were conducted using the data presented in Figure 3. (A) The first measured uniformality in the distribution of the WITS regardless of location, which returned a χ^2^ value of 1.8068 on 7 *df* (*p* = 0.9698). (B) The second tested association between the WITS and organ location by constructing an 8 × 2 contingency table comparing the total counts of each individual WITS present by organ (liver and spleen) across all mice (0.5, 6, 24, 48, and 72 h). The χ^2^ value is 2.7795, again on 7 *df* with *p* = 0.9046. The data are consistent with being uniformly spread in each organ; with no systematic differences in the probabilities of presence/absence of any particular WITS. So we conclude that the probability of colonisation of the WITS is independent of the organ.
(9 KB XLS)

Table S6 Events Included in the Stochastic Model The variables “liver_i_,” “spleen*_i_*,” and “blood*_i_*” represent the number of WITS *i* (1 ≤ *i* ≤ 8) in each compartment.
(15 KB XLS)

## Abbreviations

CFU: colony-forming units
i.v.: intravenous
p.i.: postinfection
qPCR: quantitative polymerase chain reaction
WITS: wild-type isogenic tagged strains
