# Supplementary material for: Modelling within-Host Spatiotemporal Dynamics of Invasive Bacterial Disease
Source: PLoS Biol. 2008 Apr 8;6(4):e74. doi: 10.1371/journal.pbio.0060074 (PMC2288627; doi:10.1371/journal.pbio.0060074)
Supplement: Supplementary file 1 [file pbio.0060074.sd001.doc]

**Supporting information**

**Materials and Methods – Quantitative PCR**

A standard curve was generated for each primer pair, for each different batch of QuantiTect^®^ SYBR^®^ Green used. The standard curve was calculated from four biological repeats of independent PCR premixes, analysed over two rotor runs per primer pair. Each biological repeat consisted of two samples per dilution of standard DNA. Each standard curve, per primer pair, was calculated over the range ~10^7^ to 10^1^ copies of DNA/reaction. The number of copies of DNA/reaction was calculated using the equation (Mass of DNA x Avagadro’s constant)/(Average molecular weight of a deoxynucleotide base pair x number of bases in the genome). The average molecular weight of a deoxynucleotide base pair was taken to be 660 daltons, with genome length taken to be 4.9 x 10^6^ bases (the genome sequence of the parent strain was not available when the project was started; however the sequence of SL1344 is now available at (<http://www.sanger.ac.uk/Projects/Salmonella/>)). The standard curve equation, Conc. = 10^(A(CT) + B)^, links the concentration of DNA to the number of thermocycles (CT) required to reach the fluorescence threshold at which point individual amplification reactions contain identical amounts of DNA. The values of the unknown parameters A and B can be estimated by fitting a simple linear regression model to Log_10_(conc.) against CT value, however CT values generated from repeated rotor runs can only be directly compared if the same fluorescence threshold is used for each run [1]. For each standard the Rotor-Gene software (version 6.0.14) calculates the threshold that corresponds to the values of A and B that provided the best-fitting line through the data points. For each primer pair, the biological repeats of the standards were re-analysed using their mean fluorescence threshold. This gave estimates of the parameters A and B, which were then averaged across the standards to give a final standard curve equation for each primer pair. These equations were then used to predict the unknown concentration of each WITS DNA in an experimental sample for each primer pair. Using simple linear regression to estimate the parameters A and B also allows confidence intervals around the mean concentrations to be derived on the Log_10_ scale.

Since it was not possible to generate a full standard curve for each rotor, instead a single regression line was fitted to the biological repeats of the standards within each primer pair, treating the observations as a random sample from a normal distribution. The variability reflected in the confidence intervals for the concentrations of DNA in each sample is then an artifact of the measurement variability of the qPCR process. Confidence intervals reflect the accuracy of the mean predicted concentrations; however if we wish to make inference about individual samples we can also generate a *prediction* *interval* from the data, giving us a measure of the degree of uncertainty surrounding the projected concentrations of WITS in individual samples. Converting these intervals from the Log_10_ back to the original scale is non-trivial however, and so instead a Bayesian approach to fitting the model was used. This technique treats all unknown parameters as random variables and generates *posterior densities* for the parameters and predictions of interest. A numerical Markov chain Monte Carlo algorithm, implemented in the open source package WinBUGS (<http://www.mrc-bsu.cam.ac.uk/bugs>) [2], was used to fit the model, and results were collated and analysed using another open source package, R (<http://www.r-project.org/>) [3].

Given a series of posterior predictive distributions for each individual sample, 95% credible intervals for the predicted concentrations can be calculated by taking the 2.5th and 97.5th percentiles in each case. Instead of measuring uncertainty in the mean estimates, this interval is generated centered about the median of the predicted concentrations, reflecting the fact that there is the same probability of lying above the interval as below it. Furthermore, we can generate posterior distributions for the proportions of each WITS present in each organ, and consequently each animal. A plot of the proportions for one experiment, with corresponding 95% credible intervals as calculated above, is shown in Figure 6.

**Protocol S1 - Details of Model constructions and statistical analyses**

We developed four mathematical frameworks to model specific aspects of the dynamics of local and systemic bacterial spread during systemic *S.* Typhimurium infections of mice. Analyses were carried out with the mathematical software Mathematica 6 (Wolfram Research Inc.) for Windows. Programming code is available from the authors upon request.

**SECTION 1: Testing for bacterial divisions during the early stages of infection**

The first aim was to assess from the data whether bacteria started dividing in the spleen and liver within the first 6 h p.i. Indeed, bacterial numbers (regardless of the WITS composition) in both organs show little variation between 0.5 and 6 h, suggesting a hypothetical dormant phase during which bacteria have not started dividing. The rationale was to combine the information on the numbers of CFU and WITS present in each organ for each individual mouse, into a probabilistic model. The null hypothesis was that random samples of bacteria from the inoculum are transferred into the spleen and liver without any division occurring. Given the number of bacteria observed in the liver and spleen of an individual mouse, (*n_L_*, *n_S_*), and given the sample size and composition (number of copies of each WITS) of the inoculum, it is possible, at least numerically, to estimate the probability of observing *w_L_* and *w_S_* WITS in the liver and the spleen respectively. If the observed frequency of WITS was significantly lower than expected given the numbers of bacteria, then we conclude that bacterial divisions must have occurred. Note that bacterial deaths, if they occur at random, should not lead to deviations from the expected distributions, as they would amount to reducing the effective sample size, therefore, we are conducting a series of one-tailed tests (Figure S1). Although it is possible to express analytically the probability to observe *w* WITS given a single sample of *n* bacteria and given the composition of the inoculum, we used Monte Carlo simulations to account for the paired samples and the random composition of the inoculum. The algorithm can be outlined as follows:

1. Set the sample sizes (*n_L_* and *n_S­_*) and the observed WITS frequencies (*w_L_* and *w_S_*) as observed in an individual mouse,
2. Iterate the following steps 10,000 times:
   - set up the inoculum composition by drawing at random the number of copies of each of the 8 WITS in a Poisson distribution with mean 11.65,
   - draw simultaneously two random samples of sizes *n_L_* and *n_S_*,
   - count the frequency of WITS present in the two samples,
3. Count the number of simulations (*S*) that raised at most *w_L_* and *w_S_* WITS in the pairs of samples.

The probability of observing at most *w_L_* and *w_S_* WITS given the sample sizes *n_L_* and *n_S­_* was therefore estimated as *S*/10,000. A low probability indicates that the null hypothesis may be invalid and that bacterial divisions may have occurred before observations were made. We applied the algorithm to all mice for which full information was available (n=20 at 0.5 h p.i. and n=19 at 6 h p.i.). At 0.5 h, the probabilities estimated for all but four mice were greater than 20%, three were between 5% and 20% and one was between 1% and 5% (Figure S2). In contrast, at 6 h p.i. 13/19 probabilities were less than 1%, three were between 1% and 5% and three were between 5% and 25% (Figure S2). This clearly indicates that bacterial divisions have occurred in most mice by 6 h p.i. By 0.5 h p.i., we cannot reject the hypothesis that no divisions have occurred, even though our model may not be very powerful in detecting them, given the high numbers of WITS found in the organs at that time. Therefore, given the quasi-stationary bacterial numbers in the presence of bacterial division, in further models, we make the assumption that bacterial divisions and deaths start as soon as bacteria enter the organs. The following sections estimate the rates of migration, division and death in the organs during the first 48 h of the infection.

**SECTION 2: Bacterial growth in the liver and spleen.**

**2a. Early phase: settling in the organs.**

We used the CFU in the organs to estimate the initial rates of transfer from the blood into the organs and the net growth rates. To estimate these two parameters in each organ (liver and spleen), we begin by assuming that they remain constant for at least 6 h, so that we can use the data from 0.5 and 6 h p.i. The dynamics of bacterial numbers *n_L_* (liver) and *n_S_* (spleen) (all WITS pooled together) can be described by the following equations:

$\begin{aligned} \begin{aligned} \left\{ \frac{\text{dn}_{L}}{\text{dt}}=r_{L1} n_{L}+n_{0} \theta_{L} e^{-\left( \theta_{L}+\theta_{S} \right)t} | \right. \end{aligned} \\ \end{aligned}$	**(S1)**

where *r_L_*_1_ and *r_S_*_1_ are the net growth rates in the liver and the spleen respectively (because of the low bacterial numbers used, we ignore any density-dependent effects on growth), *θ_L_* and *θ_S_* are the rates of transfer from the blood into the liver and the spleen, and *n*_0_ is the initial inoculum size. Note that $n_{0} e^{-\left( \theta_{L}+\theta_{S} \right)t}$ represents the expected number of bacteria still in the blood.

Equations S1 can be solved analytically:

$\begin{aligned} n_{L}\left( t \right)=n_{0} \frac{\theta_{L}}{r_{L}+\theta_{L}+\theta_{S}}\left( e^{r_{L1} t}-e^{\left( \theta_{L}+\theta_{S} \right)t} \right) \\ n_{S}\left( t \right)=n_{0} \frac{\theta_{S}}{r_{L}+\theta_{L}+\theta_{S}}\left( e^{r_{S1} t}-e^{\left( \theta_{L}+\theta_{S} \right)t} \right) \end{aligned}$	**(S2)**

and the four unknown parameters can be estimated numerically by equating *n_L_* and *n_S_* with the average experimental CFU in the liver and the spleen at 0.5 and 6 h p.i., for any given inoculum size *n_0_* (Figure S3). Within a reasonable range of values, the net growth rates show limited sensitivity to inoculum sizes, and remain negative in both organs. Point estimates of the parameters can be obtained by calculating the weighted average of the previous estimates under the assumption that *n*_0_ follows a Poisson distribution with mean *ν* = 93.22. Indeed, the expected value of *r_L_*_1_ is given by the following relation:

$$E\left[ r_{L1} \right]=\sum_{n_{0}\geq0} E\left[ r_{L1}|n_{0} \right] p\left( n_{0} \right)=\sum_{n_{0}\geq0} E\left[ r_{L1}|n_{0} \right] \frac{e^{-\nu} \nu^{n_{0}}}{n_{0}!}$$

and likewise for the other three parameters. The resulting estimates are shown in Table S4. The values of the migration rates suggest that virtually all bacteria are cleared from the bloodstream within around 4 h (*i.e*. $n_{0} e^{-\left( \theta_{L}+\theta_{S} \right)t}$ < 1), which is in line with the experimental results at 6 h: in 4/5 mice, no bacteria were recovered from the blood. The negative growth rates represent half-lives of bacteria of around 3 h in the liver and 5 h in the spleen.

**2b. Second phase: exponential growth**

It is clear that the negative growth rates estimated above are not compatible with the exponential growth observed by 24 h in both organs. This means that the growth rates must increase within the first 24 h. We assumed a single switch in the growth dynamics, from *r_L_*_1_ to *r_L_*_2_ and from *r_S_*_1_ to *r_S_*_2_, at a certain time (*χ*), the same in the liver and spleen. If *χ* < 6 h, then our previous estimates are incorrect, however, it is still possible to estimate the initial parameters, under the additional assumption that all bacteria from the inoculum have migrated into the organs by *χ*. First, we estimate the growth rates *r_L_*_2_ and *r_S_*_2_ between 6 and 24 h, by fitting exponential models to the average experimental bacterial counts at these two time points: *r_L_*_2_ = 0.089 h^-1^ (95% CI: 0.026-0.153), *r_S_*_2_ = 0.087 h^-1^ (95% CI: 0.036-0.138). We then extrapolate this exponential growth back to time *t* = *χ*, and combine the resulting theoretical bacterial counts with the observed counts at *t* = 0.5 h to estimate the parameters in equations (S2). Figure S4 shows that the estimates for the initial growth rates remain negative, and are all lower as the switch, from the early phase of bacterial decline to the second phase of exponential growth, occurs earlier.

If *χ* > 6 h, then we do the opposite: extrapolate model (S2), using parameter estimates (Protocol S1 Models Section 1), to *t* = *χ*, and combine the resulting theoretical bacterial numbers with those observed experimentally at *t* = 24 h to estimate the exponential growth rate in the second phase. Figure S5 shows the estimated growth rates *r_L_*_2_ and *r_S_*_2_ plotted against the initial inoculum size *n*_0_ and the switch time *χ*. Table S4 shows the point estimates under the assumptions that *χ* = 6 h and *n*_0_ follow a Poisson distribution. As explained in the next section, it is actually unlikely that the switch occurs later than 7 h p.i. We conclude that, during the second phase and up to 24 h, bacteria grow exponentially in both organs, at a rate at least equal to 0.087 h^-1^, corresponding to a doubling time of less than 8 h.

**SECTION 3. WITS dynamics**

We proceeded to disentangle bacterial divisions and deaths during the two phases previously identified *e.g*. whether the observed change in the net growth rate is due to an increase in division rate or a decrease in mortality. To this aim, we use the information provided by the number of WITS present in each organ at each time point in each mouse. Given that eight WITS were initially inoculated, and that these WITS behave identically and independently from each other (Table S5A and S5B), the proportion of WITS missing from an organ at a given time point provides an estimate of the probability that a single WITS is absent from the organ at that time.

Therefore, we developed another mathematical model that describes the dynamics of the numbers of copies of a single WITS in an organ (liver or spleen). This branching process includes: transfer from the blood to the organ, division and death. Because of the coupling between the blood and the organ, we must track the number of copies in both compartments simultaneously, at least during the first phase of the infection. We denote by *q^L^_m,n_*(*t*) the probability that *m* and *n* copies are present in the blood and in the liver respectively at time *t*, and by *q^S^_m,n_*(*t*) the probability that *m* and *n* copies are present in the blood and in the spleen respectively at time *t*. The division rates are named *λ_L_* and *λ_S_* and the death rates *μ_L_* and *μ_S_*. The dynamics for the liver in the early phase of infection are governed by the following equations:

$\begin{aligned} \left\{ q_{m,n}^{L^{'}}\left( t \right)=\mu_{L1} \left( n+1 \right) q_{m,n+1}^{L}\left( t \right)-\left[ n \lambda_{L1}+n \mu_{L1}+m \left( \theta_{L}+\theta_{S} \right) \right] q_{m,n}^{L}\left( t \right) | \right.\left\{ +\left( n-1 \right) \lambda_{L1} q_{m,n-1}^{L}\left( t \right)+\left( m+1 \right)\left[ \theta_{L} q_{m+1,n-1}^{L}\left( t \right)+\theta_{S} q_{m+1,n}^{L}\left( t \right) \right] ; n>0 | \right.\left\{ q_{m,0}^{L^{'}}\left( t \right)=\mu_{L1} q_{m,1}^{L}\left( t \right)-\left[ m \left( \theta_{L}+\theta_{S} \right) \right] q_{m,0}^{L}\left( t \right)+\left( m+1 \right) \theta_{S} q_{m+1,0}^{L}\left( t \right) | \right. \end{aligned}$	**(S3)**

The equations for the spleen can be obtained by swapping ‘*L*’ and ‘*S*’. Since we have already estimated the migration rates and the net growth rates *r_L_*_1_ = *λ_L_*_1_ – *μ_L_*_1_ and *r_S_*_1_ = *λ_S_*_1_ – *μ_S_*_1_, we only need to estimate the death rates in each organ. Given the uncertainty on the duration of the initial phase, it would have been preferable to fit the model to the data at the earliest time point, *i.e*. 0.5 h p.i. However, at this early stage, bacteria have only just started to enter the organs and undergo deaths and divisions, so this data point conveys little information on death rates. We confirmed numerically that the probability of an individual WITS being absent from either organ at *t* = 0.5 h, using equations (S3), varies very little over a wide range of parameter values (data not shown). Therefore we based our estimates on the observed proportions of WITS missing from the liver or the spleen at 6 h, under the assumption that the early phase lasts at least 6 h. The fitting procedure consisted of numerical integrations of equations (S3), varying the death rate *μ_L_*_1_ until the estimated probability that the WITS is absent from the liver at *t =* 6 h matched the experimental proportion of WITS missing from the liver at the same time (0.47). The probability is obtained by summing *q^L^_m,_*_0_ over *m* ≥ 0. We then repeated the analysis for the spleen (observed proportion of WITS missing from the spleen: 0.35). We thus obtained the following estimates: λ_L1_ = 0.38 h^-1^, μ_L1_ = 0.64 h^-1^, λ_S1_ = 0.44 h^-1^, μ_S1_ = 0.59 h^-1^.

We verified numerically that the probabilities of absence at 0.5 h predicted from the model fell within the 95% confidence intervals for the observed proportions of WITS missing. Figure S6 summarizes our results, and illustrates a further prediction based on this model: if we extrapolate the probability of absence in either the liver or the spleen beyond 6 h, based on the previous estimates, the value continues to increase and rapidly (within ~7 h) exceeds the upper limit of the 95% confidence interval from the data at 24 h p.i. So, if we assume that the first phase lasts more than 7 h, there must be a mechanism to reduce the probability of absence of an individual WITS in either organ. However, as explained in the main text (Figures 2C, 3 and 4), a more detailed examination of the data reveals that the WITS composition of the two organs remains different until at least 24 h. If mixing occurred between 6 and 24 h, we would expect the majority of WITS to be present in both organs at 24 h, which is not the case. The data indicate that mixing starts between 24 and 48 h. Therefore, we conclude that the initial phase of extinction, characterized by relatively high rates of division and death, must stop at the latest 7 h p.i. Should it stop before 6 h, then our estimates would be altered (see Table S4). However, based on our previous analyses (Protocol S1 Models Section 2.b), we are confident that our estimates are minimum values, and that both the division and death rates must all be higher as the early phase is shorter. This uncertainty does not affect our general conclusions.

We now turn to the second phase of infection, and use the observed proportions of missing WITS, together with the net growth rate estimates obtained previously, to estimate the division and death rates. Since bacteria are no longer in the blood, equations (S3) can be greatly simplified. Let *p^L^_n_*(*t*) be the probability of having *n* copies of a given strain in the liver at time *t*, the model now reads:

$\begin{aligned} \left\{ p_{n}^{L^{'}}\left( t \right)=\mu_{2}^{L}\left( n+1 \right)p_{n+1}^{L}\left( t \right)-\left( \mu_{2}^{L}+\lambda_{2}^{L} \right)n p_{n}^{L}\left( t \right)+\lambda_{2}^{L} \left( n-1 \right) p_{n-1}^{L}\left( t \right) ; n\geq1 | \right. \end{aligned}$	**(S4)**

and likewise for the spleen, by replacing ‘*L*’ with ‘*S*’. We set the initial conditions at *t = χ* by extrapolating the model given by equations (S3).

Based on the observed proportions of missing WITS at 24 h (0.5 in the liver and 0.37 in the spleen), we obtained the following estimates, assuming that the switch occurs at t = 6 h: λ_L2_ = 0.107 h^-1^, μ_L2_ = 0.017 h^-1^, λ_S1_ = 0.097 h^-1^, μ_S1_ = 0.009 h^-1^. Given the very low estimates for the death rates, it is likely that almost no bacterial mortality occurs after 6 h. We also note that the estimated division rates are lower than those in the first phase, which suggests that the early bactericidal process is replaced by a bacteriostatic process. Although our estimates are based on the assumption that the switch between the two phases occurs at 6 h p.i., our analyses show that (i) it cannot occur after 7 h, and (ii) if it occurs before 6 h, the contrast between the parameter values of the two phases would be even greater.

**SECTION 4. Full stochastic model**

**4a. Numerical insights into the latest phase of infection (after 24 h)**

Up to this point, our analyses have dealt with the first 24 h of the infection. By 48 h, the two sub-populations in the liver and spleen start mixing as the infection becomes systemic. As a result, the branching process described by equations (S4) would need to account for this coupling. Given the high numbers of bacteria involved, this approach is not practical. However, we can use the bacterial counts (regardless of WITS composition) in the organs and the blood at 48 h to estimate the late growth rate and the rate of transfer from the organs into the blood.

First, the exponential growth rate between 24 and 48 h, based on the experimental data, is around 40% higher than those between 6 and 24 h. By regression, we obtained the following estimates (valid after 24 h): *r_L_*_3_ = 0.13 h^-1^ and *r_S_*_3_ = 0.12 h^-1^. Then we consider the exchange of bacteria. We make the simplifying assumptions that (i) the rates of transfer from the blood into the organs, *θ_L_* and *θ_S_*, have not changed since the early phase, and (ii) the rate of transfer from the organs into the blood *θ_B_* is the same for the liver and the spleen. We describe the late dynamics as follows:

$\begin{aligned} \left\{ \frac{\text{dn}_{L}}{\text{dt}}=\left( r_{L3}-\theta_{B} \right) n_{L}+\theta_{L}n_{B} | \right.\left\{ \frac{\text{dn}_{S}}{\text{dt}}=\left( r_{S3}-\theta_{B} \right) n_{S}+\theta_{S}n_{B} | \right. \end{aligned}$	**(S5)**

where *n_B_* is the number of bacteria in the blood. Experimentally, the extrapolated, average number of bacteria in the blood 48 h p.i. was ~12 CFU. By combining equations (S1) for the first 6 h, independent exponential growths in each organ between 6 and 24 h, and equations (S5) from 24 h on, we estimated that the rate of transfer from the organs to the blood is around *θ_B_* = 0.005 h^-1^. We obtained a very similar estimate if we assumed that this mixing only starts after 36 h.

**4b. Stochastic simulations**

We now complete our modeling of the within-host spatiotemporal dynamics of an acute murine *S. enterica* infection with a stochastic model that encompasses all the processes previously analysed over the first 48 h of the infection. Our parameter estimations relied on average values from the experiments (CFU and numbers of WITS) but ignored the variability between individual mice. This variability can have three main sources: intrinsic differences between mice (which would amount to different parameter values), variations in the initial conditions (inoculum size and composition), or stochastic variations caused by the low numbers of bacteria involved. By running stochastic simulations, we can reproduce the latter two sources of variability. Furthermore, we gain additional information about the dynamics, in particular regarding the co-localisation of WITS in the liver and spleen, and the relative frequencies of the different WITS. Because this information is, to some extent, available from experimental data but was not used to estimate parameters, we can use it to validate our model further.

The stochastic model is based on a method by Gillespie [4]. It keeps track of 24 variables, representing the number of copies of each of the eight WITS in the blood, liver and spleen. For each WITS, we included eight possible events (deaths, divisions and transfers), listed in Table S6. We used the parameter values previously estimated and changed their values at three set time points: (i) at t=6 h, change in division and death rates, (ii) at t=24 h, increase in division rates, (iii) at t=36 h, the transfer from the organs to the blood is activated. The main outputs of the 1,000 simulations are shown in the main text (Figures 2B and 2C). In general, we observe a good correlation between the simulations and the experimental results, suggesting that we have captured a large part of the observed variability. There is still a noticeable discrepancy at 0.5 h, when the experimental data (CFU and WITS numbers) are more variable than the simulations. This could be due to heterogeneities among mice or among experiments or, possibly, to mechanistic detail not included in the model. The stochastic simulations, as well as the qPCR in the experiments, report on the number of copies of each WITS present in an organ at a given time. Therefore, we also analysed the proportions of the WITS in the organs, both from experimental data (main text Figure 6) and from simulations (Figure S7). Within each organ, we observe a relative homogeneity of all WITS at 0.5 h p.i., then strong heterogeneity at 6 and 24 h p.i. with several WITS often missing. Finally, distributions at 48 h p.i. exhibit an intermediate pattern, with one strain still constituting around a half of the bacterial composition in each organ.

**References**

1. Rutledge RG, Côté C (2003) Mathematics of quantitative kinetic PCR and the application of standard curves. Nucl Acids Res DOI: 10.1093/nar/gng093.
2. Spiegelhalter D, Thomas A, Best N, Lunn D (2003) WinBUGS User Manual, Version 1.4. <http://www.mrc-bsu.cam.ac.uk/bugs/welcome.shtml>.
3. R Development Core Team, *R: A language and environment for statistical computing* (R Foundation for Statistical Computing, Vienna, Austria. ISBN 3-900051-07-0, URL [http://www.R-project.org](http://www.R-project.org/), (2006).
4. Gillespie DT (1977) Exact stochastic simulation of coupled chemical reactions. J Phys Chem 81: 2340-2361.
5. Hanahan D (1983) Studies on transformation of *Escherichia coli* with plasmids. J Mol Biol 166: 557-580.
6. Donnenberg MS, Kaper JB (1991) Construction of an *eae* deletion mutant of enteropathogenic *Escherichia coli* by using a positive-selection suicide vector. Infect Immun 59: 4310-4317.
7. Hoiseth SK, Stocker BA (1981) Aromatic-dependent *Salmonella typhimurium* are non-virulent and effective as live vaccines. Nature 21: 238-239.
8. Hautefort I, Proença MJ, Hinton JCD (2003) Single-copy green fluorescent protein gene fusions allow accurate measurement of *Salmonella* gene expression in vitro and during infection of mammalian cells. Appl Environ Microbiol 69: 7480-7491.
9. Yanisch-Perron C, Vieira J, Messing J (1985) Improved M13 phage cloning vectors and host strains: nucleotide sequences of the M13mp18 and pUC19 vectors. Gene 33: 103-119.
10. Chang ACY, Cohen SN (1978) Construction and characterization of amplifiable multicopy DNA cloning vehicles derived from the P15A cryptic miniplasmid. J Bacteriol 134: 1141-1156.
11. Scholz O, Thiel A, Hillen W, Niederweis M (2000) Quantitative analysis of gene expression with an improved green fluorescent protein. p6. Eur J Biochem 267: 1365-1370.
12. Philippe N, Alcaraz J-P, Coursange E, Geiselmann J, Schneider D (2004) Improvement of pCVD442, a suicide plasmid for gene allele exchange in bacteria. Plasmid 51: 246-255.
13. Mo E, Peters SE, Willers C, Maskell DJ, Charles IG (2006) Single, double and triple mutants of *Salmonella enterica* serovar Typhimurium *degP* (*htrA*), *degQ* (*hhoA*) and *degS* (*hhoB*) have diverse phenotypes on exposure to elevated temperature and their growth in vivo is attenuated to different extents. Microb Pathog 41: 174-182.
